# Mental health screening tools in correctional institutions: a systematic review

**DOI:** 10.1186/1471-244X-13-275

**Published:** 2013-10-29

**Authors:** Michael S Martin, Ian Colman, Alexander IF Simpson, Kwame McKenzie

**Affiliations:** 1 Department of Epidemiology and Community Medicine, University of Ottawa, 451 Smyth Road, Ottawa, Ontario K1H 8M5, Canada; 2 Centre for Addiction and Mental Health, 1001 Queen Street West, Toronto, Ontario K1H 8M5, Canada; 3 Department of Psychiatry, University of Toronto, 1001 Queen Street West, Toronto, Ontario M6J 1H4, Canada

**Keywords:** Mental health, Screening, Inmates, Corrections, Prison, Jail

## Abstract

**Background:**

Past studies have identified poor rates of detection of mental illness among inmates. Consequently, mental health screening is a common feature to various correctional mental health strategies and best practice guidelines. However, there is little guidance to support the selection of an appropriate tool. This systematic review compared the sensitivity and specificity of mental health screening tools among adult jail or prison populations.

**Methods:**

A systematic review of MEDLINE and PsycINFO up to 2011, with additional studies identified from a search of reference lists. Only studies involving adult jail or prison populations, with an independent measure of mental illness, were included. Studies in forensic settings to determine fitness to stand trial or criminal responsibility were excluded. Twenty-four studies met all inclusion and exclusion criteria for the review. All articles were coded by two independent authors. Study quality was coded by the lead author.

**Results:**

Twenty-two screening tools were identified. Only six tools have replication studies: the Brief Jail Mental Health Screen (BJMHS), the Correctional Mental Health Screen for Men (CMHS-M), the Correctional Mental Health Screen for Women (CMHS-W), the England Mental Health Screen (EMHS), the Jail Screening Assessment Tool (JSAT), and the Referral Decision Scale (RDS). A descriptive summary is provided in lieu of use of meta-analytic techniques due to the lack of replication studies and methodological variations across studies.

**Conclusions:**

The BJMHS, CMHS-M, CMHS-W, EMHS and JSAT appear to be the most promising tools. Future research should consider important contextual factors in the implementation of a screening tool that have received little attention. Randomized or quasi-randomized trials are recommended to evaluate the effectiveness of screening to improve the detection of mental illness compared to standard practices.

## Background

Higher rates of mental disorders have consistently been reported in correctional settings as compared to the general public [1-3]. Offenders with mental illness are more likely to engage in institutional violence and rule infractions [4], especially those with psychotic or depressive symptoms [5]. Similarly, offenders with mental illness are less likely to be released on parole or other forms of discretionary release [6] and may be more likely to have their community supervision revoked [7]. Two meta-analyses showed that interventions for offenders with mental illness may be effective at improving outcomes while incarcerated [8] and at preventing further crime [9]. However, past studies have found poor identification of offenders with mental illness for treatment services. Teplin [10] found that only 32.5% of inmates with severe mental illness were detected at intake. However, this same study noted mental health needs were more likely to be identified among those with a past psychiatric treatment (91.7% of whom were detected). Similarly, whereas 45% of those with a psychotic disorder were detected by jail personnel, only 7% of those with major depression were identified. Similar results were found in the United Kingdom by Birmingham et al [11]. In their study, 23% of those with a current mental illness were identified by prison staff. However, they did not find a higher detection rate of psychotic disorders as observed by Teplin. Findings such as these have led to the inclusion of mental health screening as a key component of a correctional mental health strategy [12-15].

Brooker et al [16] remarked that while screening tools have improved the identification of individuals with mental disorder, they tend to screen in a large number of offenders without mental health needs (i.e., false positives). It has been argued that a tiered screening system which accepts higher false positive rates is a preferred option [17,18]. However, if false positive rates are too high, this may lead to an inefficient use of scarce mental health resources [19-21]. This may result in large numbers of offenders without mental health needs receiving mental health assessments, possibly delaying treatment for those of highest need. Tensions between accurately identifying needs versus provision of treatment are intensified in jail settings (i.e., for pre-trial offenders and those serving shorter sentences) where there is less time to provide treatment than in prison settings where inmates are serving long sentences (i.e. 2 years or longer in Canada).

There is a lack of consensus about what constitutes acceptable performance for a screening tool. Possible standards that administrators could attempt to achieve include: 1) maximizing detection of mental illness regardless of false positive rates; 2) maximizing detection of mental illness while maintaining the false positive rate below a threshold; 3) minimizing the number of false positives while maintaining the false negative rate below a threshold; 4) maximizing the overall accuracy with no priority given to either type of error.

Major issues in choosing a standard are determining the most important mental health conditions to detect and what referral rate can be managed with local resources. In screening for rare but severe illness (e.g. psychosis or suicidal ideation), a two-stage screening process might be appropriate. It may be tolerable to have a high false positive rate in the first stage, followed by secondary level triage to identify those in greatest need of service [17]. In community settings, this has been challenging, with lower needs individuals using disproportionately high levels of services [22,23]. To mitigate this potential concern, adding a minimal standard for specificity might be desirable.

Where resources are more limited, efficiency may be the primary consideration. Jurisdictions with long waitlists for treatment and/or short periods of time to offer treatment may be overburdened by a screening tool which refers many inmates who do not require services. In this case, a tool with high specificity and adequate sensitivity might be preferable. Alternatively, a tool with high overall accuracy might be an option. However, if the prevalence of illness is very low, overall accuracy might be high, even if the tool identifies very few individuals with mental illness. For example, the Kessler-6 (K6), which has been widely adopted in community settings, had an overall correct classification rate of 92% at the optimal cut-off of 13. However, at this cut-off, the sensitivity was only 36% [24].

As there is little guidance to inform the selection of an appropriate mental health screening tool in correctional settings, we conducted a systematic review of existing research in the area. The review was guided by four questions: (1) what are the sensitivity and specificity of screening tools in an offender population? (2) do they perform equally well across sex and ethnicity? (3) do they perform equally well at detecting severe mental illness (e.g., psychotic disorders, bipolar disorder and major depression [13]) as compared to other mental illnesses? (4) do they perform equally well in jail or remand setting (i.e., with pre-trial detainees or offenders serving short sentences) as in prison settings (i.e., among offenders serving longer sentences)?

## Methods

The Preferred Reporting Items for Systematic Reviews and Meta-Analyses (PRISMA) statement was used to guide the conduct and reporting of our systematic review [25].

### Inclusion and exclusion criteria

We sought to identify all studies published in English or French by no later than December 2011 related to mental health screening of incarcerated individuals. The search was completed in February 2012. Studies were reviewed against four inclusion and two exclusion criteria. Inclusion criteria include: (1) the sample consisted of people 18 years of age or older who were incarcerated following a charge or conviction for a criminal offence; (2) the paper examined a systematic screening process to detect potential mental illness; (3) the criterion measure was either a validated diagnostic tool or direct clinician assessment; and (4) sufficient data were available to calculate relevant statistics to assess tool performance (i.e. sensitivity, specificity, negative/positive predictive value [NPV/PPV]). Exclusion criteria were: (1) screening for cognitive functioning, intellectual disability, substance abuse, personality disorder, suicide risk, or malingering of psychiatric symptoms; (2) screening in a forensic hospital setting in the context of a pre-trial assessment (e.g. competency to stand trial or criminal responsibility).

### Literature search

**Figure 1:**
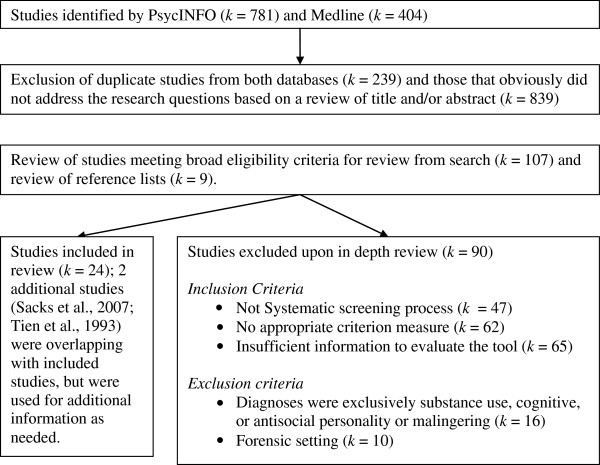
Identification and evaluation of studies relative to inclusion and exclusion criteria.

Studies were identified through a search of PsycINFO and Medline databases. The abstract and title fields were searched using a combination of terms of capture the activity of interest (i.e. screen*, assess*, identify* or *triage*), its focus (i.e. *mental health*, *psychiatric*, or *mental disorder*) and the setting (i.e. *jail*, prison*, *offender*). Terms within the three categories were joined using the OR operand and the three categories were joined using the AND operand. 781 results were returned from PsycInfo and 404 were returned from Medline. There were 946 unique results from this initial search after excluding 239 results returned from both databases. Following a review of titles and abstracts to exclude articles that obviously did not meet the research question, 107 articles remained for a complete review. Nine additional studies were identified from a review of the reference lists of these 107 articles, including one unpublished manuscript that was retrieved through a Google search [26]. One author was contacted to obtain the government report [27] containing the primary analyses that were subsequently presented in a peer reviewed manuscript [28]. Given that this review was of a descriptive nature, we erred towards being over-inclusive when reviewing papers against the criteria. Twenty-four articles met all inclusion and exclusion criteria and were included in the review (see Figure 1 for a flow-through of articles retrieved as part of this review). Two additional studies [27,29] had overlapping samples with included studies. These were used to extract additional information regarding the methods used or to retrieve data from sub-group analyses. One study [30] reported independent samples to construct and validate the Referral Decision Scale (RDS); each of which was coded as a separate study (which we refer to as the construction and the validation samples).

### Study coding

Coders used a data extraction form developed for this study to collect information about the study setting (i.e. jail, prison, health care unit, women’s institution), the sex and racial composition of the sample, the reference standard (e.g. chart diagnosis, clinician assessment, structured diagnostic interview, etc. and types of disorders covered), and statistical information regarding the performance of the screening tool (e.g. raw numbers of true and false positives and negatives, sensitivity, specificity, positive and negative predictive value (PPV and NPV), and referral rates). All studies were coded by the primary author. The remaining authors each coded approximately one third of the articles to establish inter-rater agreement. Intraclass correlation coefficients (type: consistency) exceeded .95 for continuous variables. Kappa exceeded .70 for categorical variables. Discrepancies were resolved through discussion to achieve consensus.

We calculated missing statistics where possible to address variable reporting of results. For 17 studies (63%), we calculated the referral rates using the sensitivity, specificity and either (1) the PPV and NPV or (2) the prevalence of illness. For one study [31], the calculation of referral rates highlighted a transcription error, which was corrected through contact with the author (N. Gagnon, personal communication, March 1, 2013). One study did not report sensitivity and specificity values [32]. We re-calculated sensitivity and specificity values for two other studies. For one study we calculated these values by sex from raw data provided [26]. For another study [33], data were weighted by sex, as sampling was stratified. Since no other studies accounted for over-sampling by sex, we re-calculated un-weighted statistics from the raw data provided. Overall accuracy was calculated for 13 (54%) studies and could not be calculated for another 3 (13%). PPV and NPV were calculated for 5 studies (21%). Confidence intervals were only reported in 2 studies [31,33]. To ensure consistency in calculations, we calculated confidence intervals for all sensitivity and specificity values [34].

Study quality was assessed by the primary author. Tools were rated using the QUADAS-2 [35]. The tool requires an assessment of four domains: patient selection, index test, reference standard, and flow and timing. A series of signalling questions are answered as either yes, no or unclear, and an overall assessment is made whether the domain could have introduced bias. A rating of low, high or unclear concern regarding applicability to the review topic is also made.

### Analysis

A narrative summary of findings is provided as the range of tools identified, the diverse methodologies, and the lack of replication studies precluded the meaningful use of meta-analytic techniques to aggregate the results.

## Results

### Study characteristics

Twenty-two screening tools were identified from the twenty-four studies included in the review (a list of tools and their acronyms is provided in the Additional file 1, Appendix). As seen in Table 1, the majority (*n* = 13; 54%) of research has been conducted in the United States, and almost exclusively in jail settings (*n* = 18; 75%). The majority of studies were published in peer-review journals (*n* = 20; 83%). Two doctoral theses [31,32] and two government reports [26,27] were also included in the review. Table 2, presents a summary of ratings using the QUADAS-2 tool. As seen in the table, there was only one study for which there was a low risk of bias and low concerns regarding applicability. Nine additional studies had low concerns regarding applicability, but had at least some concerns regarding potential bias. The remaining fourteen studies had concerns of potential bias and applicability to the review topic.

**Table 1:** Characteristics of studies included in the systematic review

**Characteristics**	***n *** **(%)**^**a**^	**Study references**
**Country of Study**		
US	13 (54%)	[20,29,30,32,33,36-40,42,43],[45,46]
UK	3 (13%)	[11,26,48]
Canada	4 (17%)	[19,27,28,31,41]
Australia/New Zealand	3 (13%)	[17,47,49]
Denmark	1 (4%)	[44]
**Study setting**		
Jail	18 (75%)	[11,17,19,20,26-28,30-32,37-40,43-45]
Prison	4 (17%)	[29,30,33,36,46]
Health care/psychiatric unit	2 (8%)	[36,37]
Women’s institution/unit	6 (25%)	[26,38,41,43,45,47]

**Notes**. ^a^Some percentages add up to more than 100 due to studies that were conducted in multiple settings. Studies with overlapping samples (i.e. [28] and [27], and [29] and [33]) are only counted once.

**Table 2:** QUADAS ratings of studies included in the review

	**Risk of bias**				**Applicability concerns**		
**Reference**	**Patient selection**	**Index test**	**Reference standard**	**Flow & timing**	**Patient selection**	**Index test**	**Reference standard**
[11]	L	**H**	?	L	L	**H**	L
[17]	L	L	L	L	L	L	L
[19]	L	L	L	L	L	**H**	L
[20]	**H**	L	L	L	L	L	L
[26]	L	L	L	L	L	L	L
[28]	**H**	L	L	**H**	L	L	L
[30] (Construction sample)	L	**H**	L	**H**	L	**H**	L
[30] (validation sample)	?	L	L	**H**	L	**H**	L
[32]	**H**	L	**H**	**H**	**H**	L	L
[33]	?	**H**	L	**H**	**H**	**H**	L
[31]	**H**	L	L	**H**	L	L	L
[36]	**H**	L	**H**	**H**	**H**	L	**H**
[37]	**H**	**H**	**H**	**H**	**H**	**H**	**H**
[38]	**H**	**H**	**H**	L	**H**	**H**	**H**
[39]	**H**	**H**	?	L	**H**	L	L
[40]	**H**	L	L	?	L	**H**	L
[41]	**H**	L	L	L	L	L	L
[42]	**H**	L	L	L	L	L	L
[43]	L	**H**	L	L	L	**H**	L
[44]	L	**H**	L	L	**H**	**H**	L
[45]	L	**H**	L	L	L	L	L
[47]	**H**	L	**H**	**H**	L	L	L
[48]	**H**	L	**H**	**H**	**H**	L	**H**
[49]	?	L	**H**	**H**	?	L	**H**
[46]	L	L	**H**	L	L	L	L

*L* = Low; *H* = High; ? = Unclear.

Common concerns with patient selection included sampling from populations with high rates of mental illness such as health care units and substance abuse programs [33,36,37], convenience sampling [32,38,39] and high refusal and/or drop-out rates [20,27,28,40-42]. In a number of studies [30,33,43] index tests were developed by statistically choosing a subset of items that performed best from a larger test battery, and in other studies [38,44], the index test was embedded within the diagnostic assessment. Three studies [43-45] received high risk of bias ratings for the administration of the index test due to not having a pre-specified threshold score, which may result in an over-estimation of test performance due to over-fitting [35]. A number of studies relied on chart information as a reference standard [36,46,47], which may result in misclassification. Flow and timing issues were due to the administration of the reference test predominantly [20,31,42], or exclusively [48,49] to those who screened positive, without weighting or other statistical adjustment as was done in two studies [17,19]. In other studies, the timing between the screening test and reference standard was lengthy (e.g. up to one month) [33], or the reference standard may have been known prior to screening [36,37].

### Performance of screening tools

Data from each individual study reviewed is provided in Additional file 1. Only six tools have published replication studies with independent samples: the Brief Jail Mental Health Screen (BJMHS), the Correctional Mental Health Screen for Men (CMHS-M), the Correctional Mental Health Screen for Women (CMHS-W), the England Mental Health Screen (EMHS), the Jail Screening Assessment Tool (JSAT), and the RDS. We focus primarily on these tools throughout the results section. Below we summarize possible uses of each of the six tools with replication studies to achieve the four performance standards proposed above. This is followed by results of the performance of tools for different demographic groups and correctional institutions.

### Brief Jail Mental Health Screen

The BJMHS generally had a sensitivity of approximately 60 to 65%. As exceptions to this, its sensitivity was only 34% [95% CI 47-48%] in a New Zealand study [17], and in one study [20] the sensitivity for women was 46% [95% CI 24-58%]. In one study where the standard cut-offs were not used [43], the sensitivity of the BJMHS was considerably higher, ranging from 82-95% depending on the breadth of disorders included in the case definition, and the choice of cut-off. At these lower cut-off scores that achieved higher sensitivity, there was a significant drop in the specificity of the BJMHS (ranging from 30 to 60%). In most studies, the overall accuracy was in the range of 65-75%. As the exception to this, the use of lower cut-offs with men, resulted in slightly lower overall accuracy (i.e. 58%) [43]. Given comparable overall accuracy, the less stringent cut-offs for the BJMHS that were statistically selected by Ford and colleagues may warrant further consideration as they had similar overall accuracy, but with fewer missed cases of mental illness.

### Correctional Mental Health Screen for Men

At its recommended cut-off of 6 or more items, the CMHS-M had a sensitivity of 74%, 95% CI [65-82%] in the development study [43] and 70%, 90% CI [56-81%] in the replication study [45] for the detection of an Axis I or II disorder. Lowering this cut-off to 4 or 5 might be considered by those prioritizing detection of mental illness regardless of the false positive rates, as these cut-offs achieved sensitivity of 80%, 95% CI [67-89%] and 89%, 95% CI [77-95%] respectively in the validation study sample. The decrease to a cut-off of 5 may be particularly appealing as the overall accuracy was slightly higher (79% versus 77% at a cut-off of 6) in the validation study.

### Correctional Mental Health Screen for Women

At its recommended cut-off of 5 or more items, the CMHS-W had a sensitivity of 65%, 95% CI [52-76%], in the development study [43] and 64%, 95% CI [51-75%], in the replication study [45] for the detection of an Axis I or II disorder. Lowering this cut-off to 3 might be considered by those prioritizing detection of mental illness regardless of the false positive rates, as this cut-off achieved a sensitivity of 85%, 95% CI [74-92%], in the validation study sample. However, this lowered cut-off results in a sharp increase in the false positive rate, with a specificity of 49%, 95% CI [34-64%]. A cut-off of 4 achieved a better balance of sensitivity (74%, 95% CI [62-83%]) and specificity (72%, 95% CI [56-84%]), with a similar overall accuracy (73%) to the recommended cut-off score (75%).

### England Mental Health Screen

The EMHS achieved perfect sensitivity in a small pilot study for men over the age of 21 and for women, although the sensitivity was only 50% for the small subsample of 18-21 year old males [26]. In an study [11] using a highly similar four-item tool, a sensitivity of 76%, 95% CI [67-83%] was reported. In a replication study in New Zealand [17], however, the sensitivity of the EMHS was only 42%, 95% CI [38-56%]. Overall accuracy for the EMHS was above 80% for the small pilot study. In the two larger studies, the overall accuracy was 60% in the New Zealand study, whereas it was 74% in the early study in England.

### Jail Screening Assessment Tool

Performance of the JSAT was somewhat more variable across studies, which may reflect the use of structured professional judgement to make referral decisions. In the development study [27,28], the JSAT achieved a sensitivity of 84%, 95% CI [65-94%] among men, with a specificity of 67%, 95% CI [54-74%]. On replication among a small sample of women [41], the tool performed comparably, with a slight decrease in sensitivity (75%, 95% CI [47-91%]) and a slight gain in specificity (71%, 95% CI [47-87%]). In a subsequent replication with male offenders [31] the JSAT sensitivity ranged from 38 to 50% depending on the breadth of disorders included in the case definition. A structured scoring model was proposed in this study, which would have achieved a sensitivity ranging from 67 to 72% depending on the breadth of disorders included in the case definition.

### Referral Decision Scale

As the oldest of the screening tools considered in the review, the RDS has the most extensive body of research. However, the BJMHS was developed to address limitations of the RDS, most notably concerns with the naming of the subscales corresponding with specific diagnostic categories. Veysey and colleagues noted that the RDS lacked specificity to distinguish the three categories of diagnoses (psychotic, bipolar, and major depressive disorders), and cautioned against the use of the tool due to the potential for results to be misinterpreted [37]. In the majority of studies with the general offender population [39,43,47], the RDS had high sensitivity, with low specificity. However, the study authors [30] and one other study [40] reported strong sensitivity (70% or above) and specificity (80% or above).

### Tools without replication studies

Of the tools with single studies, few appeared to perform sufficiently well to justify their implementation. The K6 and GHQ-28 may warrant further investigation in settings where the five replicated tools do not perform as well as desired given their widespread use in community and other settings [38,44]. However, neither tool performed better than the five previously mentioned tools in the initial study. The sensitivity of the K6 among women was between 58 and 69% using the pre-specified case criterion (although a restricted analysis using only those in the top quartile of symptom severity resulted in a sensitivity of approximately 80%). At the cut-point with the highest overall accuracy, the GHQ-28 had a sensitivity of 65%, 95% CI [54-75%] and a specificity of 69% 95% CI [60-77%]. The The New York State Brief Screening Tool (NYS BST) performed well for women in particular in a small study [36], with a sensitivity of 88%, 95% CI [60-97%] and a specificity of 84%, 95% CI [58-95%]. Given that a number of tools appear to perform worse among women inmates, this tool may warrant a more rigorous evaluation in a general offender population as opposed to a health care setting.

### Performance by sex

Two tool developers explored the need for sex-specific screening tests [42,43]. While items related to Post Traumatic Stress Disorder and anxiety were added to the BJMHS in an attempt to improve performance for women, the CMHS male (CMHS-M) version contains four additional items as compared to its female counterpart (CMHS-F). Steadman et al. found that the additional items did not increase performance of the BJMHS, and argued that the original version performed adequately in the second sample of women studied [42]. However, as the sensitivity was only 61%, 95% CI [49-72%] in this second study, others have argued that the BJMHS has not been adequately validated for use among women offenders [38]. The CMHS appears to perform slightly better among men than among women. Lowering the cut-off to 3 or 4 might be preferable to achieve acceptable sensitivity for women using the CMHS-W as discussed previously. The JSAT also had a slight decrease in sensitivity (75%; 95% CI [47-91%]) in a small study with women offenders [41] compared to the original research on the tool [27,28], with a similar specificity (71%; 95% CI [47-87%]). However, there was an even larger decrease in sensitivity (50% for severe mental illness; 95% CI [31-69%]) upon replication with male offenders, unless a scoring algorithm (sensitivity for severe mental illness = 67%; 95% CI [47-82%]) was used in place of structured professional judgment [31].

While the sensitivity of the NYS BST was approximately 20% higher for women (88%, 95% CI [60-97%]) than for men (67%, 95% CI [21-94%]), there is a lack of statistical power to determine whether this difference is simply the result of sampling error or a true difference in performance of the tool [36]. The RDS had high sensitivity in two studies with women [43,47], with lower specificity. It should be noted that these two studies used different cut-off scores from the traditional RDS scoring. Earthrowl et al. [47] used a cut-off of 3 on any scale, and Ford et al [43] used a cut-off of any 2 items. In both studies, referral rates exceeded 60%. Of the studies among men using the RDS some found slightly worse performance among men particularly in terms of specificity [19,37,43]. Others [30,40] found stronger performance of the RDS among men, particularly in terms of specificity.

The Co-Occurring Disorders Screening Instrument for Mental Disorder (COSDI-MD) and Co-Occurring Disorders Screening Instrument for Severe Mental Disorder (COSDI-SMD) performed comparably for men and women [29]. Unsurprisingly, the four tools (the Global Appraisal of Individual Needs Short Screener [GSS], Global Appraisal of Individual Needs Short Screener – Internal Disorder Screener [GSS-IDS], Mental Health Screening Form [MHSF], and the Mini-International Neuropsychiatric Interview – Modified [MINI-M]) from which the COSDI items were selected performed similarly among both men and women. Performance was also similar for men and women on the EMHS [26], in a small sample of 30 women.

### Performance by race/ethnicity

Few studies reported performance of tools by race. We have not reproduced the analyses by combination of sex and race presented by Ford and colleagues [43] for space reasons. They suggested comparable performance of the CMHS across races for both men and women, other than a suggestion to consider a lower cut-off score to improve the sensitivity of the tool for white women. Nonetheless, in their replication study [45] this recommendation was not pursued. The only other study to compare performance by race [33], found comparable performance of the COSDI-MD and COSDI-SMD among White, Black and Latino offenders. While not a direct test of performance in different racial/ethnic groups, two studies [17,31] failed to replicate the performance of the BJMHS and the EMHS in countries with high rates of indigenous inmates (New Zealand and Canada). In New Zealand [17], the BJMHS and EMHS lacked sensitivity in general (34%, 95% CI [30-38%], but had high specificity (86%, 95% CI [83-88%]), although as discussed below performance differed by disorder. Conversely, in the Canadian study [31], while the sensitivity of the BJMHS was similar to studies in the United States at approximately 65% in all cases, the specificity was considerably lower (i.e. 59%, 95% CI [47-69%] as compared to 76%, 95% CI [69-82%] and 84%, 95% CI [77-88%] in the original American studies [20,42].

### Performance by disorder

Few studies compared the performance of tools to detect various disorders. Evans et al [17] reported that the majority of false negatives using the EMHS and BJMHS were depressive disorders, whereas the tools missed very few cases of psychosis. The CMHS-M and CMHS-W [45], JSAT [31], and K6 [38] performed comparably across a range of diagnostic categories. The COSDI-SMD generally performed poorly, although it was more sensitive to severe mental illness (ranging from 50 to 59%) than to any axis I or II disorder (ranging from 36 to 41%) [33].

### Performance by correctional facility

Only four studies included prison populations (one of which was restricted to those in health care units [36]). The COSDI-MD, COSDI-SMD (and the tools from which these items were drawn – the MHSF, MINI-M and GSS), the MCMI-III, the NYS BST and the RDS are the only tools to be tested in a prison setting. Of these tools, only the RDS has been tested in both jail and prison settings. While the RDS had a relatively high sensitivity (79%, 95% CI [70-86%]) and specificity (99%, 95% CI [98-99%]) in a prison setting [30] as compared to other studies of the RDS, this study was the original cross-validation by the developers, which relied on a secondary data set. Replications in jail settings have had variable results, creating challenges determining whether there are differences in performance across settings for the RDS.

## Discussion

Our review identified a number of screening tools in the literature. However, the paucity of replication studies and study quality issues for a number of tools limit conclusions regarding their application. The BJMHS, the CMHS-M, the CMHS-W, the EMHS, the JSAT, and the RDS have been best studied. Given that the BJMHS was developed to address limitations of the RDS, we would discourage adoption of the RDS. However, the remaining five tools are recommended as first options for implementation, as the majority of studies have supported their use.

Whereas the BJMHS, CMHS-M and CMHS-W and EMHS are brief tools (i.e. 5 minutes or less) that can be administered by health or custodial staff, the JSAT is completed by nursing or psychology staff, and requires 20-30 minutes to complete. Only two studies included in this review compared these tools against one another. Evans et al compared the BJMHS and the EMHS, and found that they had roughly comparable performance [17]. Ford et al [43] found higher accuracy of the CMHS tools compared to the BJMHS and RDS, except for Black women. A recent study [50] in a police jail found comparable performance between the JSAT and the BJMHS.

### Contextual factors

Our review identified important contextual considerations for those selecting a tool. For example, both the BJMHS and the EMHS performed well in initial studies. However, in validation studies in Canada [31] and New Zealand [17] their performance decreased considerably, in particular in the detection of major depression [17]. Gagnon [31] and Evans et al [17] noted that differences between countries in access to health care might influence referral rates on tools such as the BJMHS and EMHS which include past psychiatric treatment items. Furthermore, both countries have relatively large Indigenous populations who have relatively less utilisation of mental health services in the community [51]. As both the BJMHS and EMHS include items regarding mental health treatment history, poorer performance in ethnically diverse populations may reflect their lack of access to health care in the community [51], or cultural differences in interpreting the meaning of constructs and tools to measure them [52]. A recent study [53] found lower referral rates among Black and Latino inmates screened with the BJMHS. Black and Latino inmates had less prior service utilization, items which result in automatic referral. The EMHS relies entirely on historical variables, whereas the BJMHS, the CMHS and the JSAT all include items regarding history and current symptoms. Thus the EMHS may be less sensitive to mental illness if inmates have low rates of past psychiatric treatment, similar to the previous findings of Teplin [10].

Staff characteristics, skills, and training also appear to be important factors. Steadman et al [20,42] found higher referral rates when screening was completed by a female as compared to a male staff member. They also found that many false negative cases were inmates who disclosed more information to health care professionals than they did to correctional officers. Steadman et al [42] noted that correctional officers felt a need for training on establishing trust and eliciting information, and that they noted challenges asking questions related to current symptoms.

### Limitations

This study is limited by the lack of replication studies of otherwise well designed tools. There have been considerable reductions in performance in the replication of some tools, therefore limiting our ability to draw conclusions about many tools reviewed. While we have attempted to include all relevant literature, it remains possible that we were unable to access or locate additional work – particularly studies in which tools performed poorly.

The lack of trials evaluating screening tools limits our ability to assess the improvements in detection rates following the introduction of a mental health screening tool. In their development study, Steadman and colleagues acknowledged that the BJMHS performed worse for women offenders, but noted that it represented an improvement over previous screening results [20]. While the argument supports the use of the tool, it was based on the results of Teplin [10] from approximately twenty years earlier. It is possible that detection would have improved since this time without screening given increased attention to mental illness in corrections. While not always feasible, an experimental or quasi-experimental design (e.g. randomized controlled trials, cluster randomized trials, stepped wedge, or time-series designs) should be used to compare detection rates prior to and following implementation of screening.

## Conclusions

Screening is a critical component to a correctional mental health strategy, and there appear to be some improvements in screening tools in recent years. Five tools with replicated results warrant consideration for implementation. A small number of tools that have been less extensively studied may also warrant further research. We have suggested four potential standards that could be used to determine what adequate performance of a screening tool means within each specific context. There are a number of factors that may impact the performance of screening tools such as sex, race/ethnicity/culture, jail versus prisons, country factors (e.g. availability of services in the community), and staff qualifications and training that have received minimal attention in the literature. An increased understanding of these factors is needed to inform more accurate, cost-effective, and feasible mental health screening.

## Abbreviations

PPV: Positive predictive value; NPV: Negative predictive value; BJMHS: Brief jail mental health screen; CMHS-M: Correctional mental health screen for Men; CMHS-W: Correctional mental health screen for Women; COSDI-MD: Co-occurring disorders screening instrument for mental disorder; COSDI-SMD: Co-occurring disorders screening instrument for severe mental disorder; EMHS: England mental health screen; GHQ-28: General health questionnaire (28 item); GSS: Global appraisal of individual needs short screener; GSS-IDS: Global appraisal of individual needs short screener - internal disorder screener; JSAT: Jail screening assessment tool; K6: Kessler 6; MHSF: Mental health screening form; MINI-M: Mini international neuropsychiatric interview – modified; NYS BST: New York State brief screening tool; RDS: Referral decision scale.

## Competing interests

The authors declare that they have no competing interests.

## Authors’ contributions

MM conceived of the study, drafted the data collection protocols, conducted the statistical analyses and drafted the manuscript. IC, AIFS, and KM contributed to the development of the data collection protocols and analysis plan, coded eligible studies, and revised drafts of the manuscript. All authors read and approved the final version of the manuscript.

## Pre-publication history

The pre-publication history for this paper can be accessed here:

http://www.biomedcentral.com/1471-244X/13/275/prepub

## Supplementary Material

Additional file 1 **Summary table of research on mental health screening tools in correctional settings.** Refer to the appendix at the end of the table for definitions of acronyms and variables. Click here for file
