# Supplementary material for: Mental health screening tools in correctional institutions: a systematic review
Source: BMC Psychiatry. 2013 Oct 29;13:275. doi: 10.1186/1471-244X-13-275 (PMC4231452; doi:10.1186/1471-244X-13-275)
Supplement: Additional file 1 — Summary table of research on mental health screening tools in correctional settings. Refer to the appendix at the end of the table for definitions of acronyms and variables. [file 1471-244X-13-275-S1.docx]

# Additional File 1. Summary table of research on mental health screening tools in correctional settings. Refer to the appendix at the end of the table for definitions of acronyms and variables.

| Tool | Study | Setting | Population | *N* | Cut-off | % referred | Criterion | % cases | Sensitivity | Specificity | Overall accuracy |
| --- | --- | --- | --- | --- | --- | --- | --- | --- | --- | --- | --- |
| **Tools with replication studies** | | | | | | | | | | | |
| BJMHS | [20] | Jail | Female; half AA | 146 | 2 symptoms or one service utilization item | 35 | SMI (C) | 42 | .46 [.34, .58] | .73 [.63, .81] | 62% |
|  | [42] | Jail | Female; majority AA | 256 | 2 symptoms or one service utilization item | 34 | SMI (C) | 24 | .61 [.49, .72] | .75 [.69, .81] | 72% |
|  | [43] | Jail | Female; majority white | 101 | Any 1 item | 85 | I or II (C) | 60 | .95 [.86, .98] | .30 [.18, .45] | 69% |
|  |  |  |  |  | Any 2 items | 69 | I (C) | 43 | .90 [.78, .96] | .46 [.34, .59] | 65% |
|  |  |  |  |  |  |  | I or II (C) | 60 | .88 [.77, .94] | .60 [.45, .74] | 77% |
|  | [20] | Jail | Male, half AA | 211 | 2 symptoms or one service utilization item | 35 | SMI (C) | 27 | .66 [.53, .76] | .76 [.69, .82] | 73% |
|  | [42] | Jail | Male, majority AA | 205 | 2 symptoms or one service utilization item | 24 | SMI (C) | 16 | .64 [.47, .78] | .84 [.77, .88] | 80% |
|  | [17] | Jail | Male; 42% white; 34% Maori; majority are remand cases | 530 | 2 symptoms or one service utilization item | 23 | SMI (C) | 46 | .34 [.30, .38] | .86 [.83, .88] | 62% |
|  | [43] | Jail | Male; half white; 35% black | 201 | Any 2 items | 53 | I (C) | 17 | .82 [.66, .91] | .53 [.45, .60] | 58% |
|  |  |  |  |  | Any item | 70 | I or II (C) | 50 | .92 [.85, .96] | .41 [.32, .51] | 58% |
|  | [31] | Jail | Male | 106 | 2 symptoms or one service utilization item | 47 | I (C) | 35 | .68 [.52, .80] | .64 [.52, .74] | 65% |
|  |  |  |  |  |  |  | I & SUD (C) | 27 | .66 [.47, .80] | .60 [.49, .70] | 61% |
|  |  |  |  |  |  |  | SMI (C) | 23 | .67 [.47, .82] | .59 [.47, .69] | 60% |
| BJMHS-R | [42] | Jail | Female; majority AA | 258 | 2 | 41 | SMI (C) | 24 | .65 [.53, .76] | .66 [.59, .72] | 66% |
|  |  |  | Male, majority AA | 206 | 2 | 33 | SMI (C) | 16 | .67 [.50, .80] | .73 [.66, .79] | 72% |
| CMHS-W | [43] | Jail | Female, majority white | 101 | 1 | 94 | I or II (C) | 60 | .98 [.91, 1.0] | .13 [.06, .27] | 64% |
|  |  |  |  |  | 2 | 87 | I (C) | 43 | .98 [.89, 1.0] | .21 [.12, .33] | 54% |
|  |  |  |  |  | 5 | 45 | I or II (C) | 60 | .65 [.52, .76] | .85 [.70, .93] | 73% |
|  |  |  |  |  | 6 | 39 | I (C) | 43 | .63 [.48, .76] | .79 [.67, .88] | 72% |
|  | [45] | Jail | Female, approximately 40% each white and black, and 20% Latin | 100 | 1 | 96 | I or II (C) | 61 | .98 [.91, 1.0] | .08 [.03, .21] | 63% |
|  |  |  |  |  | 2 | 84 | I or II (C) | 61 | .93 [.84, .97] | .33 [.20, .49] | 70% |
|  |  |  |  |  | 3 | 72 | I or II (C) | 61 | .85 [.74, .92] | .49 [.34, .64] | 71% |
|  |  |  |  |  | 4 | 58 | I or II (C) | 61 | .74 [.62, .83] | .72 [.56, .84] | 73% |
|  |  |  |  |  | 5 | 42 | I or II (C) | 61 | .64 [.51, .75] | .92 [.79, .97] | 75% |
|  |  |  |  |  | 6 | 32 | I or II (C) | 61 | .48 [.36, .60] | .92 [.79, .97] | 65% |
|  |  |  |  |  | 7 | 15 | I or II (C) | 61 | .23 [.14, .35] | .97 [.86, .99] | 52% |
|  |  |  |  |  | 8 | 2 | I or II (C) | 61 | .03 [.01, .11] | 1.0 [.91, 1.0] | 41% |
| CMHS-M | [43] | Jail | Male, majority white | 201 | 6 | 66 | I or II (C) | 50 | .74 [.65, .82] | .75 [.66, .82] | 50% |
|  |  |  |  |  | 7 | 35 | I (C) | 17 | .68 [.51, .81] | .72 [.65, .78] | 71% |
|  | [45] | Jail | Male, approximately 40% each white and black, and 20% Latin | 106 | 1 | 91 | I or II (C) | 43 | 1.0 [.92, 1.0] | .15 [.08, .26] | 52% |
|  |  |  |  |  | 2 | 86 | I or II (C) | 44 | .98 [.89, 1.0] | .23 [.14, .35] | 56% |
|  |  |  |  |  | 3 | 74 | I or II (C) | 44 | .91 [.79, .96] | .40 [.29, .53] | 62% |
|  |  |  |  |  | 4 | 62 | I or II (C) | 43 | .89 [.77, .95] | .60 [.47, .71] | 73% |
|  |  |  |  |  | 5 | 51 | I or II (C) | 45 | .80 [.67, .89] | .78 [.66, .87] | 79% |
|  |  |  |  |  | 6 | 41 | I or II (C) | 44 | .70 [.56, .81] | .83 [.71, .90] | 77% |
|  |  |  |  |  | 7 | 33 | I or II (C) | 43 | .63 [.49, .75] | .90 [.80, .95] | 78% |
|  |  |  |  |  | 8 | 20 | I or II (C) | 44 | .44 [.31, .58] | .98 [.91, 1.0] | 74% |
|  |  |  |  |  | 9 | 10 | Axis I or II (C) | 44 | .24 [.14, .38] | 1.0 [.94, 1.0] | 67% |
|  |  |  |  |  | 10 | 9 | I or II (C) | 43 | .20 [.11, .34] | 1.0 [.94, 1.0] | 65% |
|  |  |  |  |  | 11 | 4 | I or II (C) | 43 | .09 [.04, .21] | 1.0 [.94, 1.0] | 61% |
|  |  |  |  |  | 12 | 1 | I or II (C) | 43 | .02 [.00,.11] | 1.0 [.94, 1.0] | 57% |
| EMHS | [26] | women inst. | female sample | 30 | 1 | 57 | SMI (L) | 37 | 1.0 [.74, 1.0] | .68 [.46, .85] | 80% |
|  |  | Jail | male sample | 90 | 1 | 29 | SMI (L) | 19 | 1. 0 [.82, 1.0] | .88 [.78, .93] | 90% |
|  |  | Jail | young offender (18-21) male sample | 30 | 1 | 20 | SMI (L) | 7 | .50 [.09, .91] | .82 [.64, .92] | 80% |
|  | [17] | Jail | Male; 42% white; 34% Maori; majority are remand cases | 530 | 1 | 33 | SMI (C) | 47 | .42 [.38, .56] | .75 [.72, .78] | 60% |
|  | [11] | Jail | all male | 534 | 1 | 38 | I (L) | 19 | .76 [.67, .83] | .71 [.67, .76] | 74% |
|  | [48] | Jail | all male | 43 | 1 | 33 | I or II (NR) | NR | Unable to calculate – only assessed 41 positive screens out of 201 total offenders screened. PPV can be estimated at .23 as 10/41 assessed had a diagnosis | | |
| JSAT | [41] | Women inst. | female sample | 29 | SPJ | 48 | I (NR) | 41 | .75 [.47, .91] | .71 [.47, .87] | 72% |
|  | [31] | Jail | male sample | 106 | SPJ | 27 | I (C) | 35 | .43 [.28, .59] | .81 [.70, .89] | 68% |
|  |  |  |  |  |  |  | I and SUD (C) | 27 | .38 [.23, .56] | .77 [.66, .85] | 66% |
|  |  |  |  |  |  |  | SMI (C) | 23 | .50 [.31. .69] | .79 [.69, .87] | 73% |
|  |  |  |  |  | BPRS ≥4, mental health issue or placement recommendation | 43 | I (C) | 35 | .70 [.54, .83] | .69 [.58, .78] | 70% |
|  |  |  |  |  |  |  | I and SUD (C) | 27 | .72 [.54, .85] | .69 [.58, .78] | 70% |
|  |  |  |  |  |  |  | SMI (C) | 23 | .67 [.47, .82] | .65 [.54, .74] | 65% |
|  | [27, 28] | Jail | male sample | 127 | SPJ | 43 | SMI (C) | 20 | .84 [.65, .94] | .67 [.57, .75] | 70% |
| RDS | [47] | Women inst. | female; 67% Maori or Maori/Mixed; 28% White | 131 | 3 on any scale | 60 | SMI (L) | 22 | .86 [.69, .95] | .47 [.38, .57] | 56% |
|  |  |  |  |  | 3 on B | 14 | BPD (L) | Unreported – PPV = .22 | | | |
|  |  |  |  |  | 3 on D | 32 | Maj. dep (L) | Unreported – PPV = .38 | | | |
|  |  |  |  |  | 3 on S | 15 | Psy dx (L) | Unreported – PPV = .26 | | | |
|  | [19] | Jail | All male; little other information | 182 | 2 on S or 3 on D or B |  | SMI (NR) | Unreported – PPV = .35 and NPV = .92 | | | |
|  |  |  |  | 182 | 3 on B or 2 on D or S |  | SMI (NR) | Unreported – PPV = .32 and NPV = .96 | | | |
|  |  |  |  |  | 2 on D | 39 | Maj. dep (NR) | Unreported – PPV = .15 and NPV = .99 | | | |
|  |  |  |  |  | 3 on D | 20 | Maj. dep (NR) | Unreported – PPV = .19 and NPV = .98 | | | |
|  |  |  |  |  | 2 on S | 5 | Psy dx (NR) | Unreported – PPV = .33 and NPV = .97 | | | |
|  |  |  |  |  | 3 on B | 13 | BPD (NR) | Unreported – PPV = .13 and NPV = .97 | | | |
|  | [40] | Jail | Male; 82% Caucasian | 95 | 3 on B, 2 on D or S | 22 | SMI (NR) | 12 | .73 [.43, .90] | .84 [.75, .91] | 83% |
|  | [30] | Prison | Male; 51% black, 45% white | 1149 | 3 on B, 2 on D or S | 7 | SMI (L) | 8 | .79 [.70, .86] | .99 [.98, .99] | 97% |
|  |  | Jail | Male; 81% black | 728 | 1 on B | 13 | BPD (L) | 3 | .96 [.64, 1.0] | .90 [.86, .93] | 90% |
|  |  |  |  | 728 | 1 on D | 17 | Maj. dep (L) | 5 | 1. 0 [.77, 1.0] | .87 [.82, .91] | 88% |
|  |  |  |  | 728 | 1 on S | 6 | Psy dx (L) | 3 | .88 [.55, .98] | .96 [.93, .98] | 96% |
|  |  |  |  | 728 | 2 on B | 5 | BPD (L) | 3 | .92 [.59, .99] | .98 [.96, .99] | 98% |
|  |  |  |  | 728 | 2 on D | 6 | Maj. dep. (L) | 5 | .92 [.66, .98] | .98 [.96, .99] | 98% |
|  |  |  |  | 728 | 2 on S | 5 | Psy dx (L) | 4 | .67 [.38, .87] | .99 [.96, .99] | 97% |
|  |  |  |  | 728 | 3 on B | 3 | BPD (L) | 3 | .83 [.51, .96] | 1.0 [.99, 1.0] | 99% |
|  |  |  |  | 728 | 3 on D | 3 | Maj. dep.(L) | 5 | .50 [.26, .74] | 1.0 [.98, 1.0] | 97% |
|  |  |  |  | 728 | 3 on S | 2 | Psy dx (L) | 3 | .38 [.14, .68] | 1.0 [.98, 1.0] | 98% |
|  |  |  |  | 728 | 4 on B | 1 | BPD (L) | 3 | .33 [.12, .65] | 1.0 [.99, 1.0] | 98% |
|  |  |  |  | 728 | 4 on D | 1 | Maj. dep (L) | 5 | .14 [.04, .41] | 1.0 [.98, 1.0] | 96% |
|  |  |  |  | 728 | 4 on S | 0 | Psy dx (L) | 3 | .04 [.00, .36] | 1.0 [.99, 1.0] | 97% |
|  |  |  |  | 728 | 5 on B | 0 | BPD (L) | 3 | .04 [.00,.36] | 1.0 [.99, 1.0] | 97% |
|  |  |  |  | 728 | 5 on D | 0 | Maj. dep. (L) | 5 | .00 [.00,.23] | 1.0 [.98, 1.0] | 95% |
|  |  |  |  | 728 | 5 on S | 0 | Psy dx (L) | 3 | .00 [.00, .30] | 1.0 [.99, 1.0] | 97% |
|  | [43] | Jail | Female; majority white | 101 | 1 | 87 | I or II (C) | 60 | .97 [.89, .99] | .28 [.17, .43] | 69% |
|  |  |  |  | 101 | 1 | 87 | I (C) | 43 | .98 [.89, 1.0] | .21 [.12, .33] | 54% |
|  |  |  |  | 101 | 2 | 74 | I or II (C) | 60 | .90 [.80, .95] | .50 [.35, .65] | 74% |
|  |  |  |  | 101 | 2 | 74 | I(C) | 43 | .93 [.81, .98] | .40 [.28, .53] | 63% |
|  |  |  | Male; 49% White, 34% Black | 201 | 2 | 66 | I or II (C) | 34 | .87 [.77, .93] | .45 [.37, .53] | 59% |
|  |  |  |  | 201 | 2 | 66 | I (C) | 17 | .94 [.81, .98] | .40 [.33, .48] | 49% |
|  | [39] | Jail | 51% female; 79% European American | 100 | 1 on D | 79 | Maj. dep. (NR) | 13 | 1.0 [.77, 1.0] | .24 [.16, .34] | 34% |
|  |  |  |  | 100 | 2 on D | 55 | Maj. dep. (NR) | 13 | .85 [.58, .96] | .49 [.39, .59] | 54% |
|  |  |  |  | 100 | 3 on D | 31 | Maj. dep (NR) | 13 | .54 [.29, .77] | .72 [.62, .80] | 70% |
|  |  |  |  | 100 | 4 on D | 20 | Maj. dep. (NR) | 13 | .46 [.23, .71] | .84 [.75, .90] | 79% |
|  | [37] | Jail | Males with SMI; 90% male, 53% Black, 43% White | 207 | 2 on D | 74 | Maj. dep. (C) | 27 | .73 [.60, .83] | .25 [.19, .33] | 38% |
|  |  |  |  | 207 | 2 on S | 74 | Psy dx (C) | 31 | .85 [.74, .91] | .31 [.24, .39] | 47% |
|  |  |  |  | 207 | 3 on B | 84 | BPD (C) | 15 | .84 [.68, .93] | .16 [.11, .22] | 26% |
|  | | | | | | | | | | | |
| **Tools without independent replication studies** | | | | | | | | | | | |
| COSDI-MD | [33] | prison subst. abuse tx. unit | all AA; 56% male | 96 | 3 | 68 | I or II (L) | 67 | .80 [68, .88] | .56 [.39, .72] | 72% |
|  |  |  | all white; 58% male | 137 | 3 | 72 | I or II (L) | 74 | .82 [.74, .88] | .56 [.40, .70] | 75% |
|  |  |  | all Latino; 61% male | 120 | 3 | 69 | I or II (L) | 74 | .81 [.71, .88] | .64 [.47, .79] | 77% |
|  | [29] | prison subst. abuse tx. unit | female; approximately half white, 1/3 Latino, 1/5 AA | 74 | 3 | 84 | I (L) | 86 | .86 [.75, .92] | .30 [.11, .60] | 78% |
|  |  |  | male; approximately half white, 1/3 Latino, 1/5 AA | 106 | 3 | 66 | I (L) | 73 | .74 [.63, .82] | .55 [.38, .72] | 69% |
| COSDI-SMD | [33] | prison subst. abuse tx. unit | all AA population; 56% male | 96 | 2 | 30 | I or II (L) | 67 | .41 [.29, .53] | .91 [.76, .97] | 57% |
|  |  |  |  |  |  |  | SMI (L) | 30 | .59 [.41, .75] | .82 [.71, .89] | 75% |
|  |  |  | all white; 58% male | 137 | 2 | 31 | I or II (L) | 74 | .40 [.31, .49] | .94 [.82, .98 | 54% |
|  |  |  |  |  |  |  | SMI (L) | 43 | .59 [.47, .71] | .91 [.83, .96] | 77% |
|  |  |  | all Latino; 61% male | 120 | 2 | 28 | I or II (L) | 74 | .36 [.27, .46] | .97 [.84, .99] | 52% |
|  |  |  |  |  |  |  | SMI (L) | 30 | .50 [.34. .66] | .82 [.73, .89] | 73% |
|  | [29] | prison subst. abuse tx. unit | female; approximately half white, 1/3 Latino, 1/5 AA | 74 | 2 | 42 | SMI (L) | 58 | .56 [.41, .70] | .77 [.60, .89] | 65% |
|  |  |  | male; approximately half white, 1/3 Latino, 1/5 AA | 106 | 2 | 21 | SMI (L) | 30 | .50 [.34, .66] | .92 [.83, .96] | 79% |
| GHQ-28 | [44] | Jail | Remand jail in Denmark; majority in solitary confinement | 184 | 2 | 90 | I (C) | 41 | .91 [.82, .96] | .10 [.06, .17] | 43% |
|  |  |  |  |  | 3 | 79 | I (C) | 41 | .88 [.79, .94] | .28 [.20, .37] | 53% |
|  |  |  |  |  | 4 | 79 | I (C) | 41 | .88 [.79, .94] | .28 [.20, .37] | 53% |
|  |  |  |  |  | 5 | 66 | I (C) | 41 | .81 [.71, .88] | .44 [.35, .53] | 59% |
|  |  |  |  |  | 6 | 66 | I (C) | 41 | .81 [.71, .88] | .44 [.35, .53] | 59% |
|  |  |  |  |  | 7 | 54 | I (C) | 41 | .72 [.61, .81] | .58 [.49, .67] | 64% |
|  |  |  |  |  | 8 | 54 | I (C) | 41 | .71 [.60, .80] | .58 [.49, .67] | 63% |
|  |  |  |  |  | 9 | 54 | I (C) | 41 | .71 [.60, .80] | .58 [.49, .67] | 63% |
|  |  |  |  |  | 10 | 45 | I (C) | 41 | .65 [.54, .75] | .69 [.60, .77] | 68% |
|  |  |  |  |  | 11 | 45 | I (C) | 41 | .65 [.54, .75] | .69 [.60, .77] | 68% |
|  |  |  |  |  | 12 | 40 | I (C) | 41 | .59 [.48, .69] | .73 [.64, .80] | 67% |
|  |  |  |  |  | 13 | 38 | I (C) | 41 | .59 [.48, .69] | .76 [.67, .83] | 69% |
|  |  |  |  |  | 14 | 36 | I (C) | 41 | .56 [.45, .67] | .77 [.68, .94] | 68% |
|  |  |  |  |  | 15 | 35 | I (C) | 41 | .52 [.41, .63] | .77 [.68, .84] | 66% |
|  |  |  |  |  | 16 | 35 | I (C) | 41 | .52 [.41, .63] | .77 [.68, .84] | 66% |
|  |  |  |  |  | 17 | 25 | I (C) | 41 | .40 [.30, .51] | .86 [.78, .91] | 67% |
| GSS | [29] | prison subst. abuse tx. unit | female; approximately half white, 1/3 Latino, 1/5 AA | 74 | 2 | 91 | I (L) | 86 | .92 [.83, .97] | .20 [.06, .51] | 82% |
|  |  |  | male; approximately half white, 1/3 Latino, 1/5 AA | 106 | 2 | 73 | I (L) | 73 | .81 [.70, .88] | .48 [.31, .66] | 72% |
| GSS-IDS | [29] | prison subst. abuse tx. unit | female; approximately half white, 1/3 Latino, 1/5 AA | 74 | 5 | 27 | SMI (L) | 58 | .37 [.24, .52] | .87 [.71, .95] | 58% |
|  |  |  | male; approximately half white, 1/3 Latino, 1/5 AA | 106 | 5 | 14 | SMI (L) | 30 | .38 [.23, .55] | .96 [.89, .99] | 78% |
| K6 | [38] | Jail | female sample; 59% AA; diagnoses established using screening tools (PHQ, and Short Screening scale for DSM-IV PTSD) | 515 | 13 | 36 | Anx (C) | 44 | .69 [.62, .74] | .89 [.85, .93] | 80% |
|  |  |  |  |  |  | 36 | Anx (C) – top quartile | 27 | .80 [.73, .86] | .80 [.76, .84] | 80% |
|  |  |  |  |  |  | 36 | Dep (C) | 43 | .66 [.60, .72] | .87 [.82, .90] | 78% |
|  |  |  |  |  |  | 36 | Dep (C) – top quartile | 27 | .82 [.75, .88] | .81 [.77, .85] | 81% |
|  |  |  |  |  |  | 36 | PTSD (C) | 53 | .58 [.52, .64] | .89 [.85, .92] | 73% |
|  |  |  |  |  |  | 36 | PTSD(C) – top quartile | 25 | .79 [.71, .85] | .79 [.74, .82] | 79% |
|  |  |  |  |  |  |  | Dep, Anx or PTSD (C) | 66 | .53 [.48,.59] | .98 [.95, .99] | 68% |
|  |  |  |  |  |  |  | Dep, Anx or PTSD (C) – top quartile | 39 | .74 [.68, .80] | .88 [.84, .91] | 83% |
| MCMI-III | [46] | Prison | 90% male; 46% Caucasian, 30% Hispanic, 21% AA | 9468 | T score of 75 |  |  |  |  | | |
| Schizoid Scale | |  |  |  |  | 12 | MH need | 15 | Unable to calculate. Odds ratio = 2.8 | | |
| Avoidant Scale | |  |  |  |  | 25 | MH need | 15 | Unable to calculate. Odds ratio = 2.4 | | |
| Depressive Scale | |  |  |  |  | 18 | MH need | 15 | Unable to calculate. Odds ratio = 3.5 | | |
| Dependant scale | |  |  |  |  | 15 | MH need | 15 | Unable to calculate. Odds ratio = 2.7 | | |
| Histrionic scale | |  |  |  |  | 13 | MH need | 15 | Unable to calculate. Odds ratio = .8 | | |
| Narcissistic Scale | |  |  |  |  | 21 | MH need | 15 | Unable to calculate. Odds ratio = .7 | | |
| Antisocial scale | |  |  |  |  | 29 | MH need | 15 | Unable to calculate. Odds ratio = 1.5 | | |
| Sadistic scale | |  |  |  |  | 20 | MH need | 15 | Unable to calculate. Odds ratio = 1.6 | | |
| Compulsive scale | |  |  |  |  | 6 | MH need | 15 | Unable to calculate. Odds ratio = .8 | | |
| Negativistic scale | |  |  |  |  | 21 | MH need | 15 | Unable to calculate. Odds ratio = 2.1 | | |
| Self-defeating scale | |  |  |  |  | 12 | MH need | 15 | Unable to calculate. Odds ratio = 2.6 | | |
| Schizotypal scale | |  |  |  |  | 3 | MH need | 15 | Unable to calculate. Odds ratio = 4.2 | | |
| Borderline scale | |  |  |  |  | 7 | MH need | 15 | Unable to calculate. Odds ratio = 5.3 | | |
| Paranoid scale | |  |  |  |  | 7 | MH need | 15 | Unable to calculate. Odds ratio = 2 | | |
| Anxiety scale | |  |  |  |  | 38 | MH need | 15 | Unable to calculate. Odds ratio = 2.9 | | |
| Somatoform scale | |  |  |  |  | 1 | MH need | 15 | Unable to calculate. Odds ratio = 8.1 | | |
| Mania scale | |  |  |  |  | 3 | MH need | 15 | Unable to calculate. Odds ratio = 3.1 | | |
| Dysthymia scale | |  |  |  |  | 15 | MH need | 15 | Unable to calculate. Odds ratio = 3.7 | | |
| Alcohol scale | |  |  |  |  | 27 | MH need | 15 | Unable to calculate. Odds ratio = 1.4 | | |
| Drug scale | |  |  |  |  | 18 | MH need | 15 | Unable to calculate. Odds ratio = 1.6 | | |
| PTSD scale | |  |  |  |  | 6 | MH need | 15 | Unable to calculate. Odds ratio = 5.2 | | |
| Thought disorder scale | |  |  |  |  | 1 | MH need | 15 | Unable to calculate. Odds ratio = 7 | | |
| Major depression scale | |  |  |  |  | 3 | MH need | 15 | Unable to calculate. Odds ratio = 9.5 | | |
| Delusional scale | |  |  |  |  | 2 | MH need | 15 | Unable to calculate. Odds ratio = 3 | | |
| MDSIS | [39] | Jail | 51% female; 79% European American | 100 |  |  |  |  |  |  |  |
| composite score | |  |  |  | 1 | 73 | Maj. dep (NR) | 13 | 1.0 [.77, 1.0] | .31 [.22, .41] | 40% |
|  |  |  |  |  | 2 | 53 | Maj. dep (NR) | 13 | .92 [.66, .99] | .53 [.43, .63] | 58% |
|  |  |  |  |  | 3 | 39 | Maj. dep (NR) | 13 | .77 [.50, .92] | .67 [.57, .76] | 68% |
|  |  |  |  |  | 4 | 28 | Maj. dep (NR) | 13 | .62 [.36, .83] | .77 [.67, .85] | 75% |
| current depression scale | |  |  |  | 1 | 13 | Maj. dep (NR) | 13 | .46 [.23, .71] | .92 [.84, .96] | 86% |
|  |  |  |  |  | 2 | 5 | Maj. dep (NR) | 13 | .23 [.08, .50] | .98 [.92, .99] | 88% |
|  |  |  |  |  | 3 | 1 | Maj. dep (NR) | 13 | .00 [.00, .23] | .99 [.94, 1.0] | 86% |
| past mood symptoms scale | |  |  |  | 1 | 64 | Maj. dep (NR) | 13 | .92 [.66, .99] | .40 [.30, .51] | 47% |
|  |  |  |  |  | 2 | 37 | Maj. dep (NR) | 13 | .69 [.42, .87] | .68 [.58, .77] | 68% |
|  |  |  |  |  | 3 | 16 | Maj. dep (NR) | 13 | .31 [.13, .58] | .86 [.77, .92] | 79% |
| Suicide/ treatment scale | |  |  |  | 1 | 45 | Maj. dep (NR) | 13 | .63 [.37, .83] | .58 [.48, .68] | 59% |
|  |  |  |  |  | 2 | 30 | Maj. dep (NR) | 13 | .69 [.42, .87] | .76 [.66, .84] | 75% |
|  |  |  |  |  | 3 | 14 | Maj. dep (NR) | 13 | .23 [.08, .50] | .87 [.78, .93] | 79% |
| MHS-A | [32] | Jail | Male | 45 | 4 cut-offs | 84 | I or II (NR) | 80 | .94 [.82, .98] | .56 [.27, .81] | 87% |
| MHSF | [29] | prison subst. abuse tx. unit | female; approximately half white, 1/3 Latino, 1/5 AA | 74 | 3 | 93 | I (L) | 86 | .97 [.89, .99] | .30 [.11, .60] | 88% |
|  |  |  |  |  | 11 | 27 | SMI (L) | 58 | .42 [.28, .57] | .94 [.79, .98] | 64% |
|  |  |  | male; approximately half white, 1/3 Latino, 1/5 AA | 106 | 3 | 72 | I (L) | 73 | .79 [.69, .87] | .48 [.31, .66] | 71% |
|  |  |  |  |  | 11 | 15 | SMI (L) | 30 | .34 [.20, .52] | .93 [.85, .97] | 76% |
| MINI-M | [29] | prison subst. abuse tx. unit | female; approximately half white, 1/3 Latino, 1/5 AA | 74 | 5 | 81 | I (L) | 86 | .83 [.72, .90] | .30 [.11, .60] | 76% |
|  |  |  |  |  | 10 | 34 | SMI (L) | 58 | .49 [.35, .63] | .87 [.71, .95] | 65% |
|  |  |  | male; approximately half white, 1/3 Latino, 1/5 AA | 106 | 5 | 61 | I (L) | 73 | .70 [.59, .79] | .62 [.44, .77] | 69% |
|  |  |  |  |  | 10 | 24 | SMI (L) | 30 | .41 [.26, .58] | .84 [.74, .90] | 71% |
| NYS BST | [36] | Jail | Female; 69% Black, 15% Hispanic | 26 | 4 cut-offs | 41 | Need (C) | 45 | .88 [.60, .97] | 84 [.58, .95] | 73% |
|  |  |  | Male; 68% Black, 21% Hispanic | 66 |  | 28 | Need (C) | 5 | .67 [.21, .94] | .74 [.62, .83] | 74% |
| PAS | [39] | Jail | 51% female; 79% European American | 100 |  |  |  |  |  |  |  |
| Composite Score | |  |  |  | 7 | 35 | Maj dep. (NR) | 13 | 1.0 [.77, 1.0] | .75 [.65, .83] | 79% |
|  |  |  |  |  | 8 | 21 | Maj dep. (NR) | 13 | .69 [.42, .87] | .86 [.77, .92] | 84% |
|  |  |  |  |  | 9 | 19 | Maj dep. (NR) | 13 | .62 [.36, .83] | .88 [.80, .93] | 85% |
| Negative Affect | |  |  |  | 6 | 31 | Maj dep. (NR) | 13 | .92 [.66, .99] | .78 [.68, .85] | 80% |
|  |  |  |  |  | 7 | 18 | Maj dep. (NR) | 13 | .62 [.36, .83] | .89 [.81, .94] | 85% |
| PISP | [40] | Jail | Male; 82% White | 95 | NR | 8 | SMI (NR) | 12 | .45 [.21, .72] | .96 [.90, .99] | 91% |
| PISP or RDS |  |  |  |  | NR | 25 | SMI (NR) | 12 | .91 [.62, .98] | .83 [.74, .90] | 84% |
| PS – narrow | [49] | Jail | all male | 567 | 3 | 23 | Psy dx (L) | 10 | .65 [.52, .76] | .83 [.79, .86] | 86% |
| PS – broad | [49] | Jail | all male | 567 | 2 | 24 | Psy dx (L) | 10 | .75 [.62, .84] | .83 [.79, .86] | 87% |

# Appendix

Acronyms used as abbreviations for mental health screening tools

| **Acronym** | **Full Screening Tool Name** |
| --- | --- |
| BJMHS | Brief Jail Mental Health Screen |
| BJMHS-R | Brief Jail Mental Health Screen - Revised |
| CMHS-F | Correctional Mental Health Screen for Women |
| CMHS-M | Correctional Mental Health Screen for Men |
| COSDI-MD | Co-Occurring Disorders Screening Instrument for Mental Disorder |
| COSDI-SMD | Co-Occurring Disorders Screening Instrument for Severe Mental Disorder |
| EMHS | England Mental Health Screen |
| GHQ-28 | General Health Questionnaire (28 item) |
| GSS | Global Appraisal of Individual Needs Short Screener |
| GSS-IDS | Global Appraisal of Individual Needs Short Screener - Internal Disorder Screener |
| JSAT | Jail Screening Assessment Tool |
| K6 | Kessler 6 |
| MCMI-III | Millon Clinical Multiaxial Inventory–III |
| MDSIS | Mental Disability/Suicide Intake Screen |
| MHS-A | Mental Health Screen for Adults |
| MHSF | Mental Health Screening Form |
| MINI-M | Mini-International Neuropsychiatric Interview–Modified |
| NYS BST | New York State Brief Screening Tool |
| PAS | Personality Assessment Screener |
| PISP | Prisoner Intake Screening Procedure |
| RDS | Referral Decision Scale   - B = Bipolar scale - D = Depression scale - S = Schizophrenia scale |
| PS | Screening Instrument for Psychosis |

## Definitions of Criterion Variables

For all criterion measures, a notation of either L (Lifetime), C (Current) or NR (Not reported) is made in parentheses to denote the time period covered. Specific criterion measures are as follows:

- SMI = Severe mental illness (i.e. psychotic disorders, bipolar disorder, and major depression)
- Axis I = A wider range of disorders falling on Axis I than SMI. As definitions were not always clear in papers, this typically refers to SMI plus anxiety disorders, dysthymia, etc. This excludes substance abuse and paraphilias
- Axis I and II = An Axis I disorder (as defined above) or a personality disorder, other than antisocial personality disorder
- Mental health need = Non-diagnostic outcome measures, such as a clinician assessment of need for treatment, receipt of services, or a referral for mental health services.
